# Testicular morphometry as a tool to evaluate the efficiency of immunocastration in lambs

**DOI:** 10.1590/1984-3143-AR2021-0041

**Published:** 2022-06-03

**Authors:** Laiara Fernandes Rocha, Ana Lúcia Almeida Santana, Rosiléia Silva Souza, Mariana Machado-Neves, José Carlos Oliveira, Emilly Sabrina Cotrim dos Santos, Monna Lopes de Araujo, Taís Borges da Cruz, Larissa Pires Barbosa

**Affiliations:** 1 Centro de Ciências Agrárias, Ambientais e Biológicas, Universidade Federal do Recôncavo da Bahia, Cruz das Almas, BA, Brasil; 2 Departamento de Biologia Geral, Universidade Federal de Viçosa, Viçosa, MG, Brasil; 3 Escola de Medicina Veterinária e Zootecnia, Universidade Federal da Bahia, Salvador, BA, Brasil

**Keywords:** immunocastration, lambs, testicular morphometry

## Abstract

This study aimed to evaluate the efficiency of immunocastration in lambs using testicular morphometry. Thirty lambs were randomly divided into two treatments (subcutaneous administration of 1.0 mL and 0.5 mL of an anti-GnRH vaccine) and a control group (1.0 mL saline solution). The animals were vaccinated at four months of age, received a second dose 30 days later, and were slaughtered 90 days after the first vaccine dose. After slaughter, testicles were collected, and samples were removed for histological processing and evaluation of testicular morphometric parameters. Analysis of variance, Tukey’s test, and Kruskal–Wallis test were performed, with a 5% level of significance. There was a reduction in testicular weight, gonadosomatic index, seminiferous tubule diameter, germinal epithelium height, leydigosomatic index, and total tubule length. The total length per testicular gram increased in the immunocastrated group. Intrinsic spermatogenesis yield, Sertoli cell indices, and estimates of sperm and Sertoli cell production were reduced in the immunized groups (P < 0.05). The anti-GnRH vaccine in lambs at doses of 1.0 mL and 0.5 mL is sufficient to promote immunocastration, verified through severe changes in testicular morphometry from animals.

## Introduction

The anti-gonadotropin-releasing hormone vaccine (anti-GnRH) has been considered as an alternative to surgical castration (Amatayakul-Chantler et al., 2013) to improve the welfare of farm animals by eliminating the pain, stress, and infection risk associated with physical castration methods (Thompson, 2000). This vaccine can block the activity of endogenous GnRH, thereby influencing the functioning of the hypothalamic-pituitary-gonadal axis (Tesema et al., 2019). Notably, GnRH is secreted by hypothalamic neuroendocrine cells to control the secretion of follicle stimulating hormone (FSH) and luteinizing hormone (LH), which, in turn, regulates testosterone production and secretion, affecting spermatogenesis (Needham et al., 2017).

Studies evaluating the impact of immunocastration in small ruminants have found alterations in reproductive parameters, including testicular morphometry. Lents et al. (2018) reported the suppression of testicular development and sperm production in goats immunocastrated with a commercial anti-GnRH vaccine (Bopriva, Zoetis). Tesema et al. (2019) described a reduction in serum testosterone levels and histological alterations in the testes of lambs treated with the oral recombinant kisspeptin vaccine, such as the absence of spermatids in the seminiferous epithelium and reduction in tubular morphometry, confirming the halting of the spermatogenic process.

Testicular morphofunctional evaluation is known to elucidate physiological processes from the perspective of form and function (Bielli et al., 2001). The quantification of testicular histology is a valuable tool for evaluating the sperm capacity of animals under normal, pathological, or experimental conditions (Castro et al., 1997). Sperm kinetics, among other parameters, are important for characterizing testicular activity that occurs inside the seminiferous tubules (Santos et al., 2015). Therefore, the aim of this study was to evaluate the efficiency of immunocastration in lambs by using testicular morphometry.

## Methods

This study was approved by the Ethics Committee on the Use of Animals of the Federal University of Reconcavo of Bahia (UFRB), protocol number 23007.010944/2018-68.

Thirty crossbred Santa Inês lambs (4.19 ± 0.41 months old) were randomly distributed into three groups of ten. The control group, T1, control group, had 1.0 mL of saline solution administered subcutaneously to all animals; Treatments T2 and T3 received 1.0 mL and 0.5 mL of anti-GnRH vaccine subcutaneously, respectively, and a second dose was given 30 days after the first. The experimental unit was defined as one animal. A commercial vaccine, Bopriva (Zoetis, São Paulo, Brazil), was used for all immunizations, with each mL of vaccine providing 400 µg of GnRH conjugate and carrier protein.

Sixty days after the second vaccine application (D90), the animals were slaughtered and weighed, and their testes were removed. The right and left testes of each animal were weighed, and testis fragments were fixed in buffered formaldehyde for 24 h for histological processing. Testicular samples were then dehydrated in a crescent ethanol series and embedded in a 2-hydroxyethyl methacrylate solution (Historesin, Leica). Semi-serial tissue sections of 3 μm thickness were obtained using a rotary microtome (RM 2255, Leica), stained with toluidine blue-sodium borate (1%), and analyzed using a light microscope.

Morphometric and stereological analyses of the testes were performed in ten histological fields at 400x magnitude. The mean seminiferous tubule diameter (STD) was obtained by randomly measuring 30 circular tubular cross sections. These sections were also used to measure the germinative epithelium height (GEH), which was measured from the basal membrane to the tubular lumen (Morais et al., 2009). The volumetric rates between the tubular and intertubular compartments of the testes were obtained by the method described by Morais et al. (2012). The total length of the seminiferous tubules (TLST), per testis and testis gram, and gonadosomatic (GSI), leydigosomatic (LSI), and tubulosomal (TSI) indices were measured (Morais et al., 2012).

For each histological slide, ten transverse sections of seminiferous tubules at stage 1 of the cycle (SEC) were also evaluated to obtain the following variables: seminiferous epithelium cell population, intrinsic spermatogenetic yield, and Sertoli cell index, which were estimated in each animal according to the methodology described by Costa et al. (2004). The count obtained for each cell type was corrected to the average nuclear diameter and thickness of the cut, using the formula modified by Amann (1962).

The Sertoli cell population was calculated according to the production per testicle and per testicular gram of Sertoli cells and was estimated from the corrected number of nucleoli of Sertoli cells by transverse section of the seminiferous tubule and the total length of seminiferous tubules, according to the method of Hochereau-de-Reviers and Lincoln (1978).

Testicular sperm reserve (TSR) was calculated to obtain the total and per gram sperm reserve based on quantitative histology according to the method of Costa (2001). Daily sperm production (DSP) was calculated from the quantitative histology of the testicles according to the method of Amann and Almquist (1962).

The data were subjected to normality evaluation using the Shapiro–Wilk test. For variables with a normal distribution, data were analyzed using ANOVA, and means were evaluated using Tukey’s test. For non-parametric variables, the Kruskal–Wallis test was used. Results were considered significant at P < 0.05 (SPSS, 2015).

## Results

**Figure 1:**
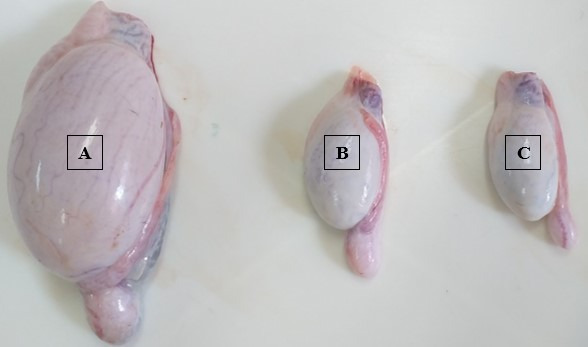
Testicular development of lambs immunocastrated or not immunocastrated with anti-GnRH vaccine. A: Control; B: 1.0 mL of the vaccine and C: 0.5 mL of the vaccine.

There was a difference in testicular weight and testicular morphometry after anti-GnRH vaccine application (P < 0.05), except for TSI (Figure 1 and Table 1). The average testis weight, GSI, STD, GEH, and LSI were within the normal ranges in the control group, whereas in both immunized groups, there was a reduction of over 50% in the analyzed variables.

**Table 1:** Testicular weight and testicular morphometry of lambs immunocastrated with the anti-GnRH vaccine.

**Variables**	**Treatments**			**P value**
	**Control**	**1.0 mL of vaccine**	**0.5 mL of vaccine**	
Morphometry 30 days after second anti-GnRH vaccine application (D90)				
TW (g)1	109.78 ± 36.99^a^	22.18 ± 11.30^b^	20.15 ± 6.71^b^	0.000
GSI (%)^1^	0.66 ± 0.17^a^	0.13 ± 0.48^b^	0.13 ± 0.03^b^	0.000
STD (µm) ^1^	204.60 ± 14.75^a^	91.59 ± 24.57^b^	87.94 ± 19.19^b^	0.000
GEH (µm) ^1^	46.71 ± 4.15^a^	11.21 ± 2.22^b^	10.13 ± 2.11^b^	0.000
LSI (%)2	0.0036 ± 0.0046^a^	0.0013 ± 0.0013^b^	0.0014 ± 0.0008^b^	0.000
TSI (%)^1^	0.28 ± 0.58	0.26 ± 0.45	0.31 ± 0.78	0.290
TLST (m)^1^	3835.7 ± 3122.3^a^	15042.2 ± 8633.8^b^	14796.4 ± 5925.2^b^	0.003
TLST/T (m/g) ^2^	11.06 ± 9.85^a^	264.92 ± 662.84^b^	335.49 ± 431.63^b^	0.000

TW: testis weight; GSI: gonadosomatic index; STD: seminiferous tubule diameter; GEH: germinative epithelium height; LSI: Leydigosomatic index; TSI: tubulosomal index; TLST: total length of seminiferous tubules; TLST/T: total length of seminiferous tubules per testicular gram. The values described correspond to the average ± standard deviation for parametric variables (^1^) and to the median ± interquartile range for non-parametric variables (^2^). Averages and medians followed by different letters in the same line are considered significantly different by the Tukey and Kruskal–Wallis tests, respectively (P < 0.05).

**Figure 2:**
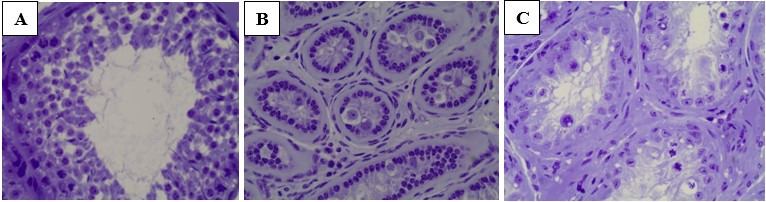
Transverse sections of seminiferous tubules from lambs immunocastrated or not immunocastrated with anti-GnRH vaccine.. A: Control; B: 1.0 mL of the vaccine and C: 0.5 mL of the vaccine. The slides were analyzed at a magnification of 400x. Immunocastration resulted in decreased thickness of the seminiferous epithelium in immunocastrated lambs (B and C) compared to that in the control group (A).

Regarding the volumetric production of testicular components, only the lumen and testicular vessels showed no differences (P > 0.05), whereas the other parameters were significantly different among the groups (P < 0.05) (Figure 2 and Table 2). The germinal epithelium, tunica propria, and connective tissue differed between the control group and T2 (1.0 mL of vaccine), while T3 (0.5 mL of vaccine) was similar to both T1 and the control groups.

**Table 2:** Volumetric proportion of the components of the testicular parenchyma of lambs immunocastrated with the anti-GnRH vaccine.

**Variables (%)**	**Treatments**			**P value**
	**Control**	**1.0 mL of vaccine**	**0.5 mL of vaccine**	
Lumen1	32.77 ± 9.57	38.34 ± 11.05	42.35 ± 16.19	0.302
Germinative epithelium2	54.77 ± 12.43^a^	40.87 ± 22.85^b^	47.42 ± 19.11^ab^	0.027
Tunica propria^1^	4.64 ± 1.53^a^	6.42 ± 1.14^b^	5.26 ± 0.43^ab^	0.011
Leydig cells^2^	1.96 ± 1.22^a^	3.43 ± 1.96^b^	2.69 ± 0.73^a^	0.000
Blood vessels^1^	0.77 ± 0.39	0.93 ± 0.16	0.94 ± 0.19	0.379
Connective tissue^1^	7.03 ± 2.89^a^	12.31 ± 3.92^b^	10.38 ± 5.11^ab^	0.027

The values described correspond to the average ± standard deviation for parametric variables (^1^) and to the median ± interquartile range for non-parametric variables (^2^). Averages and medians followed by different letters in the lines differ from each other at 5% significance, using Tukey and Kruskal–Wallis tests, respectively.

The corrected mean numbers of spermatogenetic cells and Sertoli cells found in the transverse sections of the seminiferous tubules during stage 1 of SEC were significantly different (P < 0.05) between treatments (Table 3). Evaluation of the number of cells per tubule in the transverse section is important for the quantitative analysis of spermatogenesis.

**Table 3:** Corrected cell populations of the seminiferous epithelium at stage 1 (CES) of lambs immunocastrated with the anti-GnRH vaccine.

**Variables**	**Treatments**			**P value**
	**Control**	**1.0 mL of vaccine**	**0.5 mL of vaccine**	
Spermatogonia type (A)	0.95 ± 0.12^a^	2.29 ± 0.20^b^	2.54 ± 0.53^b^	0.000
Spermatocytes in pre-leptotene (PL)	11.46 ± 3.03^a^	1.34 ± 1.23^b^	1.14 ± 1.24^b^	0.000
Spermatocytes in pachytene (PQ)	15.44 ± 3.12^a^	0.00 ± 0.00^b^	0.00 ± 0.00^b^	0.000
Rounded Spermatids (Ar)	50.29 ± 5.82^a^	0.02 ± 0.06^b^	0.00 ± 0.00^b^	0.000
Sertoli cells (S)	5.27 ± 0.79^a^	10.67 ± 1.73^b^	13.25 ± 2.64^c^	0.000

The values described correspond to the average ± standard deviation. Averages followed by different letters in the lines differ from each other at 5% significance, according to Tukey’s test.

All parameters of the intrinsic yield of spermatogenesis differed between the treatments (P < 0.05) (Table 4). The animals from the control group had spermatogenesis within the expected standards for the species and age. However, the animals that were vaccinated with 1.0 mL or 0.5 mL showed a reduction of up to 100% in spermatogenesis, indicating the effectiveness of the anti-GnRH vaccine.

**Table 4:** Intrinsic yield of spermatogenesis in lambs immunocastrated with the anti-GnRH vaccine.

**Cell type**	**Treatments (reazon)**			**P value**
	**Control**	**1.0 mL of vaccine**	**0.5 mL of vaccine**	
A:PL1	1:12.37 ± 3.92^a^	1:0.59 ± 0.54^b^	1:0.51 ± 0.55^b^	0.000
PL:PQ^1^	1:1.37 ± 0.25^a^	1:0.00 ± 0.00^b^	1:0.00 ± 0.00^b^	0.000
PQ:Ar2	1:3.16 ± 0.33^a^	1:0.00 ± 0.00^b^	1:0.00 ± 0.00^b^	0.000
A:Ar^1^	1:53.79 ± 9.91^a^	1:0.01 ± 0.03^b^	1:0.00 ± 0.00^b^	0.000

A:PL: spermatocytes in pre-leptotene/leptotene and spermatogonia type A; PL:PQ: spermatocytes in pre-leptotene/leptotene, and spermatocytes in pachytene; PQ:Ar: rounded spermatids and spermatocytes in pachytene; A:Ar: rounded spermatids, and spermatogonia type A. Values described correspond to the average ± standard deviation for parametric variables (^1^) and to the median ± interquartile range for non-parametric variables (^2^). Averages and medians followed by different letters in the lines differ from each other at 5% significance, by the Tukey and Kruskal–Wallis tests, respectively.

Sertoli cell indices were altered in lambs immunocastrated with the anti-GnRH vaccine (P < 0.05) (Table 5). Only the variable spermatogonia type A and Sertoli cells (S/A) showed no difference, because in the immunized groups, these were the only cell types present in the seminiferous tubules. Ultimately, there was a difference in the estimates of sperm production and Sertoli cells in total and per testicular gram (P < 0.05), as calculated from the histology (Table 6).

**Table 5:** Sertoli cell index of lambs immunocastrated with the anti-GnRH vaccine.

**Cell type**	**Treatments (reazon)**			**P value**
	**Control**	**1.0 mL of vaccine**	**0.5 mL of vaccine**	
S:A	1:0.18 ± 0.03	1:0.21 ± 0.03	1:0.19 ± 0.04	0.145
S:PL	1:2.27 ± 0.93^a^	1:0.13 ± 0.14^b^	1:0.09 ± 0.10^b^	0.000
S:PQ	1:3.05 ± 1.00^a^	1:0.00 ± 0.00^b^	1:0.00 ± 0.00^b^	0.000
S:Ar	1:9.84 ± 2.49^a^	1:0.00 ± 0.00^b^	1:0.00 ± 0.00^b^	0.000
S:CG	1:15.36 ± 4.40^a^	1:0.36 ± 0.16^b^	1:0.29 ± 0.10^b^	0.000

S:A: spermatogonia type A and Sertoli cells; S:PL: primary spermatocytes in pre-leptotene/leptotene and Sertoli cells; S:PQ: primary spermatocytes in pachytene Sertoli cells; S/Ar: rounded sperm and Sertoli cells; S:CG: total germ cells and Sertoli cells. The values described correspond to the average ± standard deviation. Averages followed by different letters in the lines differ from each other at 5% of significance, by the Tukey tests.

**Table 6:** Estimates of sperm production and total and per testicle gram Sertoli cells in lambs immunocastrated with the anti-GnRH vaccine.

**Variables**	**Treatments**			**P value**
	**Control**	**1.0 mL of vaccine**	**0.5 mL of vaccine**	
NSCT (×10^9^)^1^	7.05 ± 6.47^a^	55.23 ± 33.80^b^	66.72 ± 30.18^b^	0.010
NSCT/g (×10^9^)2	0.03 ± 0.049^a^	1.94 ± 6.15^b^	4.04 ± 7.61^b^	0.000
DSP (×10^9^)^2^	4.52 ± 0.97^a^	0.00 ± 0.00^b^	0.00 ± 0.00^b^	0.000
DSP/g (×10^6^)^2^	35.88 ± 8.99^a^	0.00 ± 0.00^b^	0.00 ± 0.00^b^	0.000
TSR (×10^9^)^2^	47.53 ± 10.25^a^	0.00 ± 0.00^b^	0.00 ± 0.00^b^	0.000
TSR/g (×10^6^)^2^	376.75 ± 250.19^a^	0.00 ± 0.00^b^	0.00 ± 0.00^b^	0.000

NSCT: total and per gram number of Sertoli cells; DSP: total and per gram daily sperm production; TSR: total and per gram testicular sperm reserve; the values described correspond to the average ± standard deviation for parametric variables (^1^) and to the median ± interquartile range for non-parametric variables (^2^). Averages and medians followed by different letters in the lines differ from each other at 5% significance, according to the Tukey and Kruskal–Wallis tests, respectively.

## Discussion

The reduced testis weight in immunized lambs may be associated with the neutralization of testicular function caused by the vaccine, with a consequent reduction in the testicular parenchyma. Han et al. (2015b) also observed a 50% reduction in the testis weight of immunocastrated lambs after using the anti-GnRH vaccine. Moreover, the reduced testis weight was reflected in the IGS found in this study, which was 80.3% lower in the immunized lambs than in the control lambs. The IGSs found in the control group were similar to those found by Lents et al. (2018) in 8-month-old goats.

Both 1.0 mL and 0.5 mL of anti-GnRH vaccine were capable to reduce the STD by 55.23% and 57.02% respectively, in comparison to control animals. Similarly, Han et al. (2015a), after evaluating the effect of immunocastration in rams, obtained a reduction of 35.71% in the STD of the immunized group. This concurs with Ülker et al. (2009), who reported a reduction in testicular development, as well as in the STD due to immunocastration.

The GEH response in the control group (46.71 ± 4.15µm) was similar to that found by Santos et al. (2015), when evaluating the influence of season in mixed-breed sheep (44.92 ± 9.23μm). The reduction of approximately 77% in GEH in the immunized groups in the present study reinforces the effectiveness of the vaccine in blocking the action of GnRH. From this, the production and release of the gonadotropins LH and FSH are compromised, and spermatogenesis is interrupted, consequently altering the testicular parenchyma.

The percentage of body mass allocated to Leydig cells in the control group was 0.003 ± 0.002%. According to Sarti et al. (2011), LSI is the most appropriate parameter for comparison among species, as it considers body mass and not just testicular mass. According to Vyas and Raval (2016), changes in this index indicate an increase or decrease in testosterone production. In the present study, a significant reduction in this index was observed in the immunized groups. It is likely that the vaccine inhibits GnRH activity, preventing the production and release of LH; therefore, there is no stimulation of the Leydig cells, reducing the LSI.

The total length of the seminiferous tubules found in the control group was similar to that reported by Martins et al. (2008) (3671.3 ± 1057.3 m) when evaluating sheep of undefined breed at 13 months of age. Variations in the diameter and volume occupied by seminiferous tubules in the testis interfered with this variable, but there was an increase in the immunized groups. This probably occurred because of the reduction in STD, which is justified by the decrease in GEH and the diameter of the lumen of the seminiferous tubule.

The TLST/T found for the control group was similar to that of most mammals investigated, between 10 and 20 m (França and Russell, 1998). A greater STD implied a smaller tubular length per testicular gram, as observed in the control group. The immunized groups showed higher values because the STD was lower than that recommended for the species as a result of the vaccine action, which promoted interruption of spermatogenesis and reduced STD and testicle weight.

The immunized groups had smaller proportions of germinal epithelium; however, the group that received 0.5 mL of the vaccine presented similar results to the control group, but with a significant difference in the number of sperm cells. The percentage found in the tubular compartment was similar to that reported by França and Russell (1998), who reported that seminiferous tubules account for 70%–90% of the testicular parenchyma in most mammals.

Regarding the intertubular compartment, the volumetric proportion increased in the immunized groups, but all groups remained within the range indicated in the literature. According to França and Russell (1998), this value can range from 10% to 40%. The results obtained reflect the reduction in the STD and tubular compartment, because as one component of the testicular parenchyma reduces, the other tends to increase proportionally.

The average number of type A spermatogonia per section of seminiferous tubule obtained in the control group is similar to that reported by Kilgour et al. (1998), who reported 0.91 ± 0.15 type A spermatogonia, which is within the expected range for the species. In contrast, the same authors reported a lower value (0.79 ± 0.15) for the immunized group than that obtained in this study. According to Paula (1999), the presence of this cell type, even in immunocastrated animals, occurs because these cells are self-renewing, maintain a spermatogonial reserve, and can gradually repopulate the germinal epithelium if there is cell loss.

As a result of the decrease in cell layers (as demonstrated by GEH), there was a reduction in the number of spermatogenic cells. The populations of PL, PQ, and Ar cells in the control group were lower than that reported in the literature but greater than that of the immunized groups. According to Kerr et al. (1993), spermatocytes in pachytenes and spermatids are highly sensitive to testosterone suppression. As the vaccine inhibits the activity of GnRH in the hypothalamus-pituitary-gonadal axis, reducing the production of testosterone in immunized animals, the production of cells of the spermatogenic lineage is affected.

The number of Sertoli cells was higher in the immunized groups. The population found in the control group was similar to that reported elsewhere in terms of species and age. For instance, Nazari-Zenouz et al. (2016) obtained 6.60 ± 0.22 in six-month-old sheep. Sertoli cells are more resistant to lesions capable of affecting other cell types in the seminiferous epithelium (Kumi-Diaka et al., 1983).

All variables evaluated to determine the intrinsic spermatogenetic yield had lower values in the immunized groups, with values close to zero. This response was expected based on the results of the GEH. Only the efficiency coefficient of spermatogonial mitoses (A/PL) presented a ratio above zero. This is due to some animals having spermatocytes in the PL and spermatogonia A. This result also reflects the immunocastrating effect of the vaccine, which uses the animal's own immune system to inactivate the GnRH produced. The temporary interruption of the hypothalamic-pituitary-gonadal axis through the establishment of an immune barrier neutralizes the GnRH, preventing passage from the hypothalamus to the action site in the pituitary gland, ultimately impacting the spermatogenic process (Xu et al., 2018).

Using the efficiency coefficient of spermatogonial mitoses (A/PL), it is possible to quantify the losses that occur during the spermatogonial phase. In the animals in the control group, 12.37 ± 3.92 spermatocytes in PL were produced from type A spermatogonia. França and Russell (1998) indicated that the expected value for most domestic animals is between 14.6 and 24.8.

During meiotic prophase (PL/PQ), no significant cell loss was observed in the control group, as expected, according to França and Russell (1998), from a spermatocyte in PL, a spermatocyte in PQ is produced. Likewise, it was observed that the meiotic yield (PQ/Ar) was within the normal range: for every four rounded sperm cells expected, three were formed. Losses of 21% were obtained during this phase in this study, whereas losses were generally 25% in mammals (França and Russell, 1998).

The general yield of spermatogenesis (A/Ar) is a measure of the overall efficiency of the spermatogenic process. In the present study, 78.98% of cells were lost during the entire process. Of the expected 100% (256) of rounded spermatids, 21.01% (53.79) were formed. These losses can vary from 70% to 85% in domestic mammals (França and Russell, 1998). The cell loss that occurs naturally in the spermatogenic process is probably due to density-dependent degeneration, where apoptosis is the homeostatic mechanism used to limit germ cells to a number that can be supported by available Sertoli cells (De Rooij and Lok, 1987).

Spermatogenetic efficiency can be estimated from the ratio of the germ cell population to the Sertoli cell population (Johnson et al., 2000), since Sertoli cells do not undergo numeric changes after puberty and are less vulnerable to harmful agents when compared to germ cells.

The S/PQ ratio obtained in the control group corroborates the findings of Sousa et al. (2014) in Morada Nova sheep, whereas the S/Ar ratio was higher than that reported by the authors. The total production of PQ is usually adjusted numerically to support the capacity of Sertoli cells, causing the degeneration of large numbers of cells (De Rooij and Lok, 1987). According to França and Russell (1998), the S/Ar ratio is the most important index for estimating the sperm efficiency in animals. When the relationship between these factors is strong, sperm production is also high.

The number of germ cells supported by Sertoli cells in the present study was 15.36 ± 4.40. The number of Sertoli cells per testicle is the main factor determining sperm production and testicular size (França et al., 1995).

It is common for losses to occur during spermatogenesis, mainly through apoptosis, and hormonal fluctuations in serum gonadotropin levels, with FSH secretion being the most important (Young and Nelson, 2001). In the absence of testosterone, the Sertoli cell barrier is compromised, germ cells do not complete meiosis, immature germ cells are prematurely displaced from Sertoli cells, and mature sperm are not released (Walker and Cheng, 2005). The interruption of any of these testosterone-dependent steps results in failure of spermatogenesis, causing infertility (Kerr et al., 1993).

To calculate the total and per gram number of Sertoli cells (NSCT; NSCT/g), TLST was used in addition to the number of Sertoli cells. As the TLST was high owing to variations in the diameter and volume occupied by the seminiferous tubules in the testicle, there was an increase in the immunized groups as a consequence of STD reduction, which is justified by the decrease in the GEH and lumen diameter of the seminiferous tubule.

A DSP of 4.52 ± 0.97 × 10^9^ sperm was found in the control group, a value higher than that obtained by Egu (2017) (2.60 × 10^9^ sperm). This difference may be related to the greater reproductive precocity of the studied animals. For DSP/g of testicle, 35.88 ± 8.99 × 10^9^ sperm was obtained in the control group in the present study. According to França and Russell (1998), the DSP/g of the testicle in sheep, which is considered a species with high spermatogenic efficiency, is approximately 20–30 × 10^9^ sperm. This variable is one of the most efficient parameters, which can be easily compared between species, as it eliminates the diversity in testicular weight and duration of spermatogenesis.

It is possible to quantify the potential number of sperm produced in the testes at each SEC using TSR. The average value in the control group was 47.53 ± 10.25 × 10^9^ for TSR and 376.75 ± 250.19 × 10^6^ for TSR/g. The TSR/g value was higher than that observed for most domestic animals (120–260 × 10^6^) (França and Russell, 1998).

## Conclusion

The use of the anti-GnRH vaccine in lambs at doses of 1.0 mL and 0.5 mL is efficient in promoting immunocastration, verified through significant changes in testicular morphometry from animals.
